# Ligand‐protein interactions in lysozyme investigated through a dual‐resolution model

**DOI:** 10.1002/prot.25954

**Published:** 2020-06-15

**Authors:** Raffaele Fiorentini, Kurt Kremer, Raffaello Potestio

**Affiliations:** ^1^ Max Planck Institute for Polymer Research Mainz Germany; ^2^ Physics Department University of Trento Trento Italy; ^3^ INFN‐TIFPA Trento Institute for Fundamental Physics and Applications Trento Italy

**Keywords:** coarse‐graining, dual‐resolution modeling, free energy calculation, multiscale modeling, protein‐ligand binding

## Abstract

A fully atomistic (AT) modeling of biological macromolecules at relevant length‐ and time‐scales is often cumbersome or not even desirable, both in terms of computational effort required and a posteriori analysis. This difficulty can be overcome with the use of multiresolution models, in which different regions of the same system are concurrently described at different levels of detail. In enzymes, computationally expensive AT detail is crucial in the modeling of the active site in order to capture, for example, the chemically subtle process of ligand binding. In contrast, important yet more collective properties of the remainder of the protein can be reproduced with a coarser description. In the present work, we demonstrate the effectiveness of this approach through the calculation of the binding free energy of hen egg white lysozyme with the inhibitor di‐N‐acetylchitotriose. Particular attention is payed to the impact of the mapping, that is, the selection of AT and coarse‐grained residues, on the binding free energy. It is shown that, in spite of small variations of the binding free energy with respect to the active site resolution, the separate contributions coming from different energetic terms (such as electrostatic and van der Waals interactions) manifest a stronger dependence on the mapping, thus pointing to the existence of an optimal level of intermediate resolution.

## 1 INTRODUCTION

One of the most relevant challenges of computational biochemistry and biophysics is the accurate calculation of binding free energies,^1^, ^2^, ^3^ which represents one of the key steps in the identification of pharmacological targets as well as in the development of new drugs.^4^, ^5^, ^6^ However, the large sizes of the proteins under examination (often above the hundreds of residues), as well as the necessity to screen through large datasets of potential candidate drugs they can interact with, make this effort onerous in terms of time and computational resources.

A promising way to mitigate these limitations is the use of multiple‐resolution models of the protein, that is, representations in which different parts of the molecule are concurrently described at different levels of accuracy.^7^, ^8^, ^9^, ^10^, ^11^, ^12^ The chemically relevant part of the protein, for example, the active site, is modeled at a higher level of detail, typically atomistic (AT). For the remainder, on the contrary, a simplified representation is used, where several atoms are lumped together in effective interaction sites. The working hypothesis underlying these methods is that only a relatively small part of the molecule requires an explicitly AT treatment; the remainder, in fact, is mainly responsible for large‐scale, collective fluctuations whose function‐oriented role is well recognized and prominent,^12^, ^13^, ^14^, ^15^, ^16^ however also prone to be accurately reproduced by lower‐resolution representations.^17^, ^18^, ^19^, ^20^, ^21^, ^22^ Hence, the resulting model favorably joins the accuracy of an AT description where needed and the computational efficiency of a coarse‐grained (CG) one where possible.

In order to take full advantage of the dual‐resolution approach to protein modeling, though, one has to solve a few key open issues: first, the definition of the appropriate CG model to employ in the low‐resolution part^22^, ^23^, ^24^, ^25^, ^26^, ^27^, ^28^, ^29^, ^30^; second, the coupling between high‐ and low‐resolution models, which has to be performed so as to guarantee that the observables of interest are reproduced with respect to the reference provided for example by a fully AT simulation. This issue entails a further one, namely the identification of observables apt to quantify the fidelity with which the behavior of the system is reproduced by the dual‐resolution model; third, the selection of the subpart of the molecule that *requires* a high‐resolution modeling. In the present work, we will focus specifically on this third aspect.

Various methods and approaches have been developed in the past few years to describe proteins in dual resolution.^8^, ^9^, ^10^, ^11^, ^31^, ^32^ In general, the high‐resolution part is modeled at the all‐atom level, making use of one of the several AT force fields available. The CG representations range from simple bead‐spring elastic networks^12^, ^17^, ^20^ to more sophisticated Gō‐type models.^8^ Other approaches maintain the high‐resolution description for the solute while employing a simplified model for the solvent, with varying degrees of detail depending on the specific systems and applications^33^, ^34^, ^35^, ^36^, ^37^, ^38^; among these, some treat the solvent with an adaptive resolution approach, that is, solvent molecules are AT in proximity of the solute and smoothly blend in a CG representation away from it.^39^, ^40^, ^41^, ^42^, ^43^, ^44^, ^45^, ^46^, ^47^

Recently, we have proposed a dual‐resolution model^12^ where, in the CG part, only the C_α_ carbons of the protein chain are retained and connected one with the other by harmonic bonds. This model has been employed in the present work with the aim of assessing the accuracy of a hybrid AT/CG description of a protein for binding free energy calculations. The system under examination is hen egg‐white lysozyme (HEWL) in explicit water, bound to a sugar substrate, di‐N‐acetylchitotriose. We carried out calculations of the binding free energy of the ligand in the active site, with a 2‐fold objective. In fact, not only we aimed at verifying that the computed quantity in the dual‐resolution model matches a reference, all‐atom calculation; but rather we also investigated the impact of different choices in the definition of the high‐resolution subdomain. This aspect bears the highest prominence, as it is becoming increasingly more evident that a crucial component in the construction of accurate and effective low‐resolution models for biological and soft matter systems is represented by the mapping,^12^, ^29^, ^30^ that is, the particular selection of collective variables employed to describe the system. Here, we provide novel evidence of this general property in the context of a dual‐resolution model of a biomolecule, and describe a broadly applicable strategy to tackle this issue.

## 2 METHODS

The system under examination in the present work is HEWL in aqueous solution. In this model, the binding site of the enzyme and the substrate molecule, the inhibitor di‐N‐acetylchitotriose, are represented with AT detail. The protein model employed is not adaptive, that is, the resolution of a given residue is fixed—either AT or CG—and does not change throughout a simulation. However, at difference with other works,^7^, ^8^, ^46^ several values of the number of protein residues treated at high resolution have been explored and employed in independent calculations. The impact of choosing different numbers of active site residues to model at the AT level is a central aspect of this study. The CG model employed to describe the low‐resolution part of the protein is a simple bead‐spring representation where the selected sites (namely the C_α_ atoms) are connected by elastic bonds penalizing the deviations from the distances that interacting atoms have in the reference conformation. Two values of elastic constants are employed, one for C_α_'s along the chain, and one for all other bonds. Water molecules are described in AT detail throughout the whole simulation box: the interaction with the high‐resolution part of the protein takes place through the standard all‐atom force field, while the interaction with the CG beads is mediated by a purely repulsive potential acting on the sole oxygen atom.

Hereafter we provide a detailed description of the model. We first discuss the calculation of the binding free energy Δ*G*_bind_, then we outline the dual‐resolution model and its coupling to the AT part, and finally report information about the simulation setup. Further details are made available in the Supporting Information.

### 2.1 Binding free energy calculation

One of the key points of this work is the calculation of the protein‐ligand binding free energy Δ*G*_bind_, which quantifies the affinity of a molecule toward a protein.^1^, ^2^, ^3^ As such, it plays a prominent role in the investigation of the biochemical function and activity of enzymes and similar biomolecules, and in the development of effective drugs.

Δ*G*_bind_ is defined as the difference between the free energy of the system in the configuration in which the ligand is bound to the active site (*G*_b_) and the corresponding value when the ligand is absent (*G*_ub_):

(1) $$\Delta G_{\text{bind}}=G_{b}-G_{\mathrm{ub}}.$$

This value, in the specific case under examination, varies according to the number of active site residues modeled with AT resolution, as we will see in Section 3.

The free energy difference between two states is here computed by means of thermodynamic integration (TI).^48^ Specifically, a scalar *λ* ∈ [0, 1] is defined that parameterizes the potential energy of the system as *U*_λ_(**r**) = *λU*_*A*_(**r**) + (1 − *λ*)*U*_*B*_(**r**) connecting the states *A* and *B*. The sought quantity is given by:

(2) $$\Delta G=\int_{0}^{1}{〈\frac{\partial U\left(\lambda \right)}{\partial \lambda }〉}_{\lambda }\mathrm{d\lambda}.$$

**FIGURE 1:**
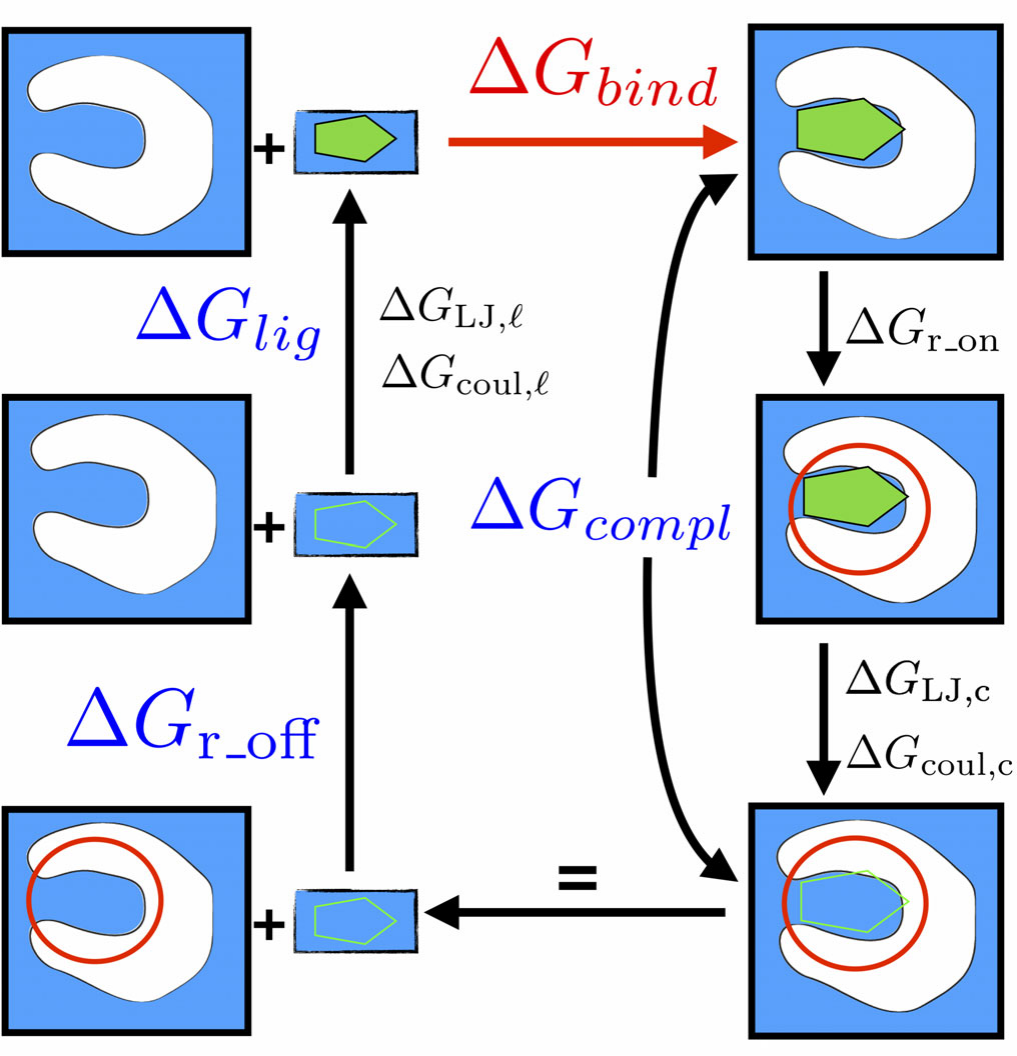
Pictorial representation of the thermodynamic cycle employed in this work. Starting from the top‐right corner of the figure, we decouple the ligand from the protein (Δ*G*_compl_, which also includes a set of restraints between ligand and protein) and subsequently introduce it in water (Δ*G*_lig_). A further step is the restraints removal (Δ*G*_r_off_) whose calculation is analytical [Color figure can be viewed at wileyonlinelibrary.com]

Since the free energy is a state function, the nature of the path is unimportant, and one can choose a thermodynamic cycle that connects the bound and unbound states through several intermediate ones, as illustrated in Figure 1. In particular, we can identify two main terms: the insertion of the ligand from vacuum to water Δ*G*_lig_, and the decoupling from the protein Δ*G*_compl_. A further step is the removal of the restraints that keep the ligand in proximity of the protein (Δ*G*_r_on_ as shown in Figure 1) during the damping of the ligand‐protein interactions, that is Δ*G*_r_off_; this latter calculation can be carried out analytically without the need to run simulations. A detailed explanation of each term and its relative alchemical changes for its calculation can be found hereafter and, in particular, in the Supporting Information in the section “Thermodynamic cycle for binding free energy.”

The binding free energy Δ*G*_bind_ is thus the algebraic sum of the previous three terms:

(3) $$\Delta G_{\text{bind}}=\Delta G_{\text{compl}}+\Delta G_{l\mathrm{ig}}+\Delta G_{r\_\mathrm{off}}.$$

According to the previous definitions of each term, neither Δ*G*_lig_ nor Δ*G*_r_off_ changes with the protein resolution: indeed, the former corresponds to the solvation free energy of the ligand, which is always treated at the AT level; likewise, the calculation of the restraint removal free energy is analytic.^3^ The only term that varies depending on the number of active site residues modeled in high resolution is the free energy change of the protein‐ligand complex between the bound state and the state where the ligand is removed, that is, the variation of Δ*G*_bind_ is equal to the variation of Δ*G*_compl_.

The alchemical change in the calculation of Δ*G*_compl_ is performed in three steps (in the following, the subscripts c and ℓ stand for complex and ligand, respectively). First, one adds a set of restraints between protein and ligand (Δ*G*_r_on_) in order to avoid the problem of the ligand leaving the binding pocket when interactions are removed. The presence of restraints is indicated in the cycle scheme of Figure 1 with a red circle: it represents the fact that the ligand is confined in a certain volume. For this work, we use the set of restraints described by Boresch.^3^ Second, Coulomb interactions are switched off (Δ*G*_coul, c_); third, the Lennard‐Jones potentials modeling van der Waals interactions are removed (Δ*G*_LJ, c_). Likewise, the alchemical change in the ligand free energy Δ*G*_lig_ is performed in two steps: first switching on Coulomb interaction (Δ*G*_coul, ℓ_), and then Lennard‐Jones (Δ*G*_LJ, ℓ_). The last contribution to the binding free energy, Δ*G*_r_off_, derives from restraint removal. These transformations are summarized in Figure 1 and Table 1. Further details can be found in the Supporting Information in the section relative to the thermodynamic cycle.

**TABLE 1:** Summary of the alchemical changes and the protein resolution dependence for each contribute of binding free energy Δ*G*_bind_

	Alchemical changes	Protein resolution dependence
Δ*G*_compl_	Δ*G*_coul,c_ + Δ*G*_LJ,c_ + Δ*G*_r_on_	Yes
Δ*G*_lig_	Δ*G*_coul, ℓ_ + Δ*G*_LJ, ℓ_	No
Δ*G*_r_off_	Analytical	No

The calculation of Δ*G*_compl_ can be carried out in two different ways, namely decoupling and annihilation. Decoupling refers to turning off the interactions between the molecule and its environment, while maintaining the potentials among atoms constituting the molecule; annihilation, on the other hand, implies turning off the interaction between the molecule and the environment *as well as* the intramolecular interaction. Here we consider the values of Δ*G* obtained through ligand decoupling, since this process is more intuitive with respect to annihilation; furthermore, the ligand is always treated at fully AT detail, therefore it is not involved in the change of free energy while varying the protein resolution. In Table 3 and Figure 6 (and with greater detail in the Supporting Information, annihilation section) we provide data showing that the values of binding free energy obtained using decoupling and annihilation are consistent within the error bars.

An important aspect that stems from Table 3 is that the largest contributions to the binding free energy come from the first two terms of Equation (3). Specifically, the insertion of the ligand in water (Δ*G*_lig_) and the decoupling of the ligand from the protein (Δ*G*_compl_) contribute to the total binding free energy with terms of the same order of magnitude, as shown in the first and second column. On the other hand, the third term of Equation (3), that is Δ*G*_r_off_, is one order of magnitude smaller than the previous two (as shown later in Section 3); however, it is not negligible for the calculation of the overall binding free energy.

### 2.2 Dual‐resolution protein model

Proteins undergo both high frequency, localized fluctuations about transient conformational substates, and slower, more global transitions between them.^49^, ^50^ In the present molecular modeling approach, those local fluctuations that can play an important role in the biological function of the protein of interest are allowed by the all‐atom description of the binding site. The set of these protein residues that are modeled with AT detail does not change during the simulation, that is, the protein has a fixed, position‐ and time‐independent dual resolution. The rest of the protein is described through a CG, lower‐resolution model. If, on the one hand, it is reasonable to expect that regions of the molecule far away from the active site have a negligible direct impact on the latter, on the other hand the collective fluctuations that they determine are important to modulate the structure of those residues involved in the binding.^22^, ^51^ Hence, to ensure the correct structure and conformational fluctuations of the binding site, it is necessary to provide a representation of the remainder of the molecule that, albeit lower‐resolution, is nonetheless capable of reproducing the appropriate large‐scale dynamics.

**FIGURE 2:**
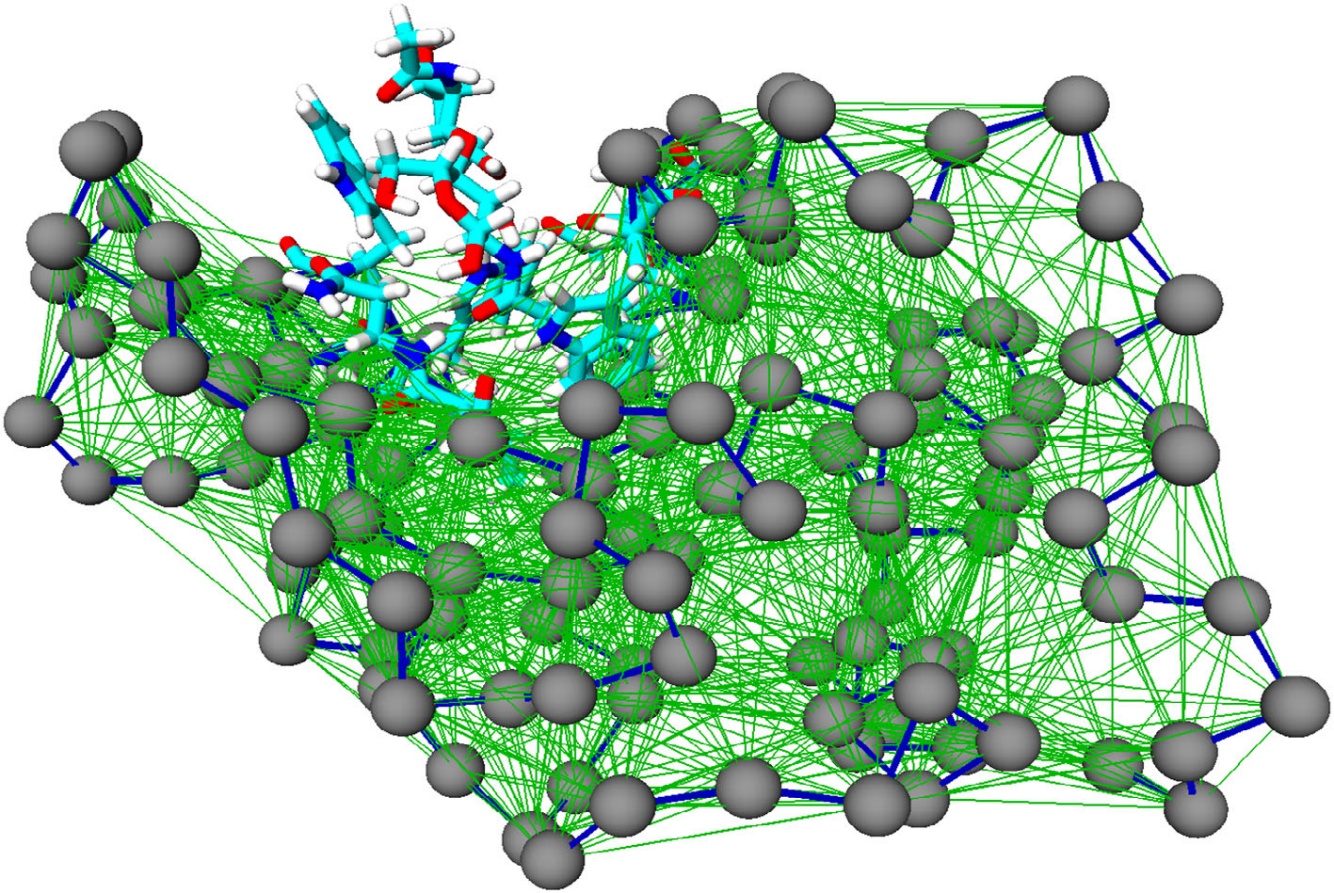
Visualization of the dual‐resolution protein.^12^ The residues included in atomistic detail are shown in red, blue, cyan, and white (O, N, C, and H atoms). The gray spheres are elastic network model nodes, the stiff backbone springs are shown as dark blue lines and all others (weaker) springs are shown in green [Color figure can be viewed at wileyonlinelibrary.com]

To describe the lower‐resolution part we thus employ an elastic network model (ENM),^12^, ^17^ in which each residue is mapped onto a bead whose position corresponds to the *C*_α_ atom in the AT description. These beads are connected by harmonic springs as shown in Figure 2.

The potential energy is given by:

(4) $$E=\sum_{i}\sum_{j}k_{\mathrm{ij}}{\left(r_{\mathrm{ij}}-r_{\mathrm{ij}}^{0}\right)}^{2}\theta \left(r_{c}-r_{\mathrm{ij}}^{0}\right)$$

with spring constants *k*_*ij*_, equilibrium distance $r_{\mathrm{ij}}^{0}$, a cutoff distance *r*_*c*_, *i* and *j* are the node index, and *θ*(*r*) is a Heaviside function taking value 1 if *r* > 0 and 0 otherwise. In this model we made use of two different elastic constants: a very stiff spring (*k*_*b*_) for consecutive beads, represented in blue in Figure 2; and a weaker spring *k*_*nb*_ for not consecutive beads whose distance in the reference (native) conformation lies below a fixed cutoff (in green).

The ENM used here is parameterized to reproduce the conformational fluctuations of the reference all‐atom model, these being quantified by the root mean square fluctuations (RMSF) of the all *C*_α_ atoms of the system.^12^ The residues in direct contact (H‐bonding or hydrophobic contact) with the substrate are modeled with all‐atom detail; in order to select the other binding site residues to be described at the AT level, we sorted them by increasing distance of their center of mass from the closest ligand atom. The solvent is treated with all‐atom detail and it surrounds the dual‐resolution protein. The water‐CG protein interaction consists in a simple excluded volume term, modeled via a Weeks‐Chandler‐Anderson (WCA) potential.^52^ The details about the procedure followed to determine the ENM elastic constants and the excluded volume interaction are provided in the Supporting Information, while the numerical values of the resulting parameters are reported hereafter.

As anticipated, the focus of the present work lies in the analysis of the impact that a modulation of the resolution of a protein in proximity of the active site can have on physical and mechanical properties of the latter, as well as on the information that the study of this impact can reveal. However, the multiresolution description can, in principle, also provide a valuable computational advantage. In fact, a dual‐resolution model can be significantly faster than the equivalent fully AT one. The speedup, which depends primarily on the fraction of atoms retained as such,^53^ is about 2 for the system investigated here: this value is relatively low, due to the fact that lysozyme, albeit a relevant, nontrivial protein, is still relatively small. In this dual‐resolution model, up to 10 residues out of 129 are described at the all‐atom level, and the degree of coarse‐graining of the low‐resolution part is not drastic (one interaction site per residue). Additionally, it has to be kept in mind that a considerable fraction (actually the majority) of the degrees of freedom of the whole setup is due to the water modeled with all‐atom detail.

A much more relevant speedup can be achieved in larger systems, for example, high molecular weight proteins, antibodies, or viral capsids, for which lower degrees of detail are allowed in the CG region. The main advantage of a dual‐resolution treatment of these macromolecules, possibly in combination with an adaptive resolution model of the solvent, is indeed that the computational gain increases with the system size, that is, precisely for those systems for which an all‐atom description becomes challenging.

### 2.3 Simulation details

The reference model is given by the 2 ns equilibrated PDB structure 1HEW in the NPT ensamble (the Parrinello‐Rahman barostat^54^ with a time constant of 2.0 ps and a pressure of 1 bar was used). Both fully AT and dual‐resolution models of HEWL are solvated in water and placed in a cubic simulation box of 7.06 nm side. The force field employed is Amber99SB,^55^ whereas the water model is TIP3P.^56^ The inhibitor, which was always AT, had GLYCAM force field parameters consistent with Amber99SB.^57^ The TI binding free energy calculation consists of three different steps: Δ*G*_compl_, Δ*G*_r_off_, Δ*G*_lig_:

1. The protein‐ligand complex free energy (Δ*G*_compl_) calculation uses 11 *λ* values per Δ*G*_restr_on, c_, 5 evenly spaced *λ* values per Δ*G*_LJ, c_ (with separation 0.20) and 15 *λ* values per Δ*G*_coul, c_, with 600 ps of simulation per *λ* in the fully AT case, and 4000 ps in the dual‐resolution case to improve the statistics.
2. The restraint removal free energy (Δ*G*_r_off_) calculation.
3. The ligand solvation free energy (Δ*G*_lig_) calculation uses 5 evenly spaced *λ* values per Δ*G*_coul, ℓ_ (with separation 0.20) and 16 *λ* values per Δ*G*_LJ, ℓ_, with 600 ps of simulation of each *λ*‐value.

In the TI, we employ the soft‐core potential of Reference 58 with parameters *α* = 0.5 and *P* = 1.0 to avoid possible singularities in the Lennard‐Jones terms from atoms overlapping during the alchemical change. The temperature is kept constant at 298 K by means of a Langevin thermostat with a friction constant *γ* = 15 ps^−1^. The integration step is 1 fs. The calculation of electrostatic interaction is performed using the reaction field method with a dielectric constant *ɛ* = 80 and a cutoff of 1.2 nm. These parameters are a good compromise between speed and accuracy, as verified in Reference 59. The SETTLE^60^ and RATTLE^61^ algorithms for rigid water and rigid bonds to hydrogen have been used. Each system is prepared using fully AT minimization with steepest descent and 6 ns of equilibration in NVT (for both ligand‐free and ligand‐bound systems). All simulations (both fully AT and dual‐resolution) are carried out with the ESPResSo++ simulation package,^62^, ^63^ in which we have implemented TI (except in case of annihilation, for which all steps are performed in both ESPResSo++ and GROMACS^64^). Some preliminary fully AT equilibration simulations use GROMACS. The error bars shown are calculated using the Student *t* at 95% confidence limit,^65^ via standard deviations obtained using block averaging in which all trajectories are divided into four blocks of equal length.

The parameterization of the dual‐resolution model is consistent with the work in Reference 12: the spring constant between consecutive *C*_α_ nodes along the backbone (*k*_b_) has a stiff value of 5 × 10^4^ kJ mol^−1^ nm^−2^, while all the other ones (*k*_nb_) have a value of 160 kJ mol^−1^ nm^−2^, until 1.2 nm as cutoff, parameterized by minimizing the average root mean square error in the *C*_α_ RMSF. Moreover, a WCA interaction is applied between *C*_α_ nodes and all solvent molecules' center of mass. In the WCA potential, *ɛ* has a value of 0.34 kJ mol^−1^ arbitrarily chosen as the value for carbon in the AT force field, and *σ*_*i*_ = *R*_*g*, *i*_ · *c* where *R*_*g*, *i*_ is the radius of gyration of a given residue *i* where *c* is the same for all amino acids. The value of *c* is tuned to give the correct bulk water density of reference for a protein‐water system. The *c* value found is 0.658. Further explanations about *c* can be found in the Supporting Information. The raw data about the simulations and analyses performed in this work are freely available on the Zenodo repository https://zenodo.org/record/3665677.

## 3 RESULTS AND DISCUSSION

We performed the calculation of Δ*G*_bind_ of lysozyme modeled in dual‐resolution, varying the number of AT residues constituting the binding site and comparing the results with a fully AT reference simulation. Recall that the binding free energy calculation consists of three steps: restraint removal, ligand Δ*G*, and ligand‐complex Δ*G*; of these, only the latter depends on protein resolution, that is, only Δ*G*_compl_ assumes different values for different numbers of active site residues described at the all‐atom level.

As explained in the previous section, the contribution coming from the restraints can be analytically computed and amounts to Δ*G*_r_off_ =  − 31.3 kJ mol^−1^. Likewise, the Coulomb and Lennard‐Jones contributions to the ligand free energy Δ*G*_lig_ are the following:

$$\begin{array}{l} \Delta G_{\text{coul},l}=-142.8\pm 1.7\;\mathrm{kJ}\;{\mathrm{mol}}^{-1}\\ \;\;\Delta G_{\mathrm{LJ},l}=-9.1\pm 6.3\;\mathrm{kJ}\;{\mathrm{mol}}^{-1}. \end{array}$$

Hence,

$$\Delta G_{\mathrm{lig}}=-151.9\pm 8.0\;\mathrm{kJ}\;{\mathrm{mol}}^{-1}.$$

**FIGURE 3:**
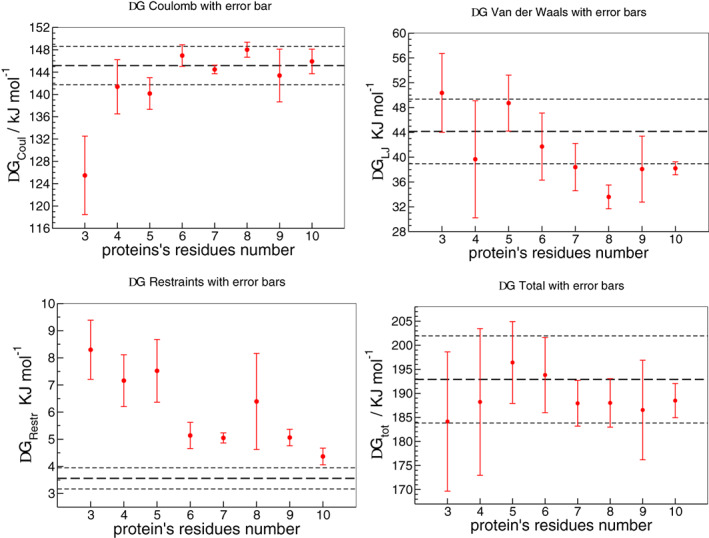
A, Coulomb; B, Lennard‐Jones; C, restraint; and D, total free energies in the protein‐ligand complex, as a function of protein's residues number included in atomistic detail in the multiresolution setup. The heavy dashed black horizontal lines are the reference values from fully atomistic simulations, and the lighter dotted black horizontal lines are the error bars for those values. These simulations use decoupling, not annihilation. Y‐axes do not cover the same energy range [Color figure can be viewed at wileyonlinelibrary.com]

The final step is the calculation of Δ*G*_compl_, whose results, including the comparison between dual‐resolution model and fully AT reference, are shown in Table 2 and illustrated in Figure 3.

**TABLE 2:** The resulting values of the complex free energy (fourth column) and its components (Coulomb, Lennard‐Jones, and restraints, respectively, in the first three columns) in fully atomistic system and varying the number of atomistic residues

At res	Δ*G*_Coul,c_	Δ*G*_LJ,c_	Δ*G*_Restr_on, c_	Δ*G*_compl_
Fully‐at	145.2 ± 3.5	44.2 ± 5.2	3.6 ± 0.4	193.0 ± 9.1
aa‐3	125.5 ± 7.0	50.4 ± 6.3	8.3 ± 1.1	184.2 ± 14.4
aa‐4	141.4 ± 4.9	39.7 ± 9.4	7.2 ± 1.0	188.3 ± 15.3
aa‐5	140.2 ± 2.8	48.7 ± 4.5	7.5 ± 1.2	196.4 ± 8.5
aa‐6	147.0 ± 1.9	41.7 ± 5.4	5.1 ± 0.5	193.8 ± 7.8
aa‐7	144.5 ± 0.8	38.4 ± 3.8	5.0 ± 0.2	187.9 ± 4.8
aa‐8	148.0 ± 1.4	33.6 ± 1.9	6.4 ± 1.8	188.0 ± 5.1
aa‐9	143.4 ± 4.7	38.1 ± 5.3	5.1 ± 0.3	186.6 ± 10.3
aa‐10	145.9 ± 2.2	38.2 ± 1.0	4.4 ± 0.3	188.5 ± 3.5

*Note*: All the values are in kJ mol^−1^ and performed with thermodynamic integration. Moreover, all simulations are carried out in ESPResSo++. In particular, for each value of *λ*, the dual‐resolution simulations with different number of atomistic residues last 4 ns; the atomistic simulation, instead, lasts 0.6 ns (600 ps).

The first three columns of the table describe the Coulomb, Lennard‐Jones, restraints contributions to free energy, respectively, while the last one corresponds to the value of the total ligand‐protein complex free energy. All the values are expressed in kJ mol^−1^. In Figure 3, the AT reference is represented with a dashed black line with its error bar. In particular, panels A‐C show the three components that contribute to the total complex free energy, reported in panel D. Looking at these values as a function of the number of all‐atom active site residues, we notice that there are important deviations of the free energy from the reference, especially in the case of 3 and 4 AT residues. On the contrary, the total value of the binding free energy agrees with the reference within the error bar in all cases.

Furthermore, we observe that the trend of free energy values, in comparison to the reference, is essentially the same: starting from 3 amino acids, it approaches the reference until reaching 6, both in its components and in total. In contrast, going from 6 to 8 AT residues the free energy value deviates from the reference, even though the total remains close to it. Finally, from 8 to 10, Δ*G* converges again. Hence, increasing the number of AT residues does not introduce necessarily an improvement of the computed free energy, at least as long as the various free energy components are considered separately.

**FIGURE 4:**
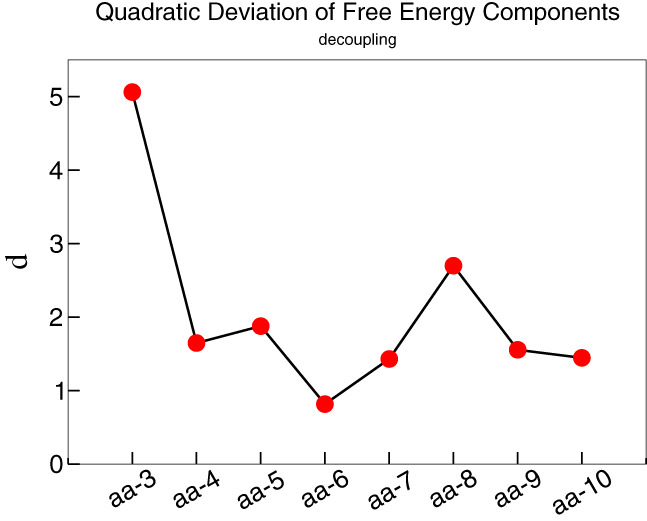
Square root of the quadratic deviation *δ* ^2^ vs the number of atomistic residues chosen. The plot shows that in the case of six atomistic residues, the value of quadratic deviation is the lowest one and hence it means that such a number leads the best result of free energy. Moreover, the black line shows the trend of free energy values as discussed in Section 3 [Color figure can be viewed at wileyonlinelibrary.com]

In order to gain further, quantitative insight into these results, we computed the quadratic deviation from the reference, *δ* ^2^, defined as:

(5) $$\delta_{i}^{2}=\delta_{i‐\text{Coul}}^{2}+\delta_{i‐\mathrm{LJ}}^{2}+\delta_{i‐\text{Restr}}^{2}={\left(\Delta G_{\text{Coul}\_i}-\Delta G_{\text{Coul}‐\mathrm{at}}\right)}^{2}+{\left(\Delta G_{\mathrm{LJ}\_i}-\Delta G_{\mathrm{LJ}‐\mathrm{at}}\right)}^{2}+{\left(\Delta G_{\text{Restr}\_i}-\Delta G_{\text{Restr}‐\mathrm{at}}\right)}^{2},$$

where the index *i* = 3, …, 10 runs over AT residues. Figure 4 reports *δ* ^2^ as a function of the number of active site amino acids modeled with AT detail.

The plot shows that the binding free energy computed in the dual‐resolution model approaches the reference as the number of AT active site residues increases, and most importantly, this trend persists for each component up to 6 residues. Beyond this value, though, the trend stops and the deviation becomes larger, peaking at 8 residues and decreasing when further AT amino acids are added. These results highlight a nonmonotonic dependence of the free energy on the mapping, that is, the number of retained AT residues. If, on the one hand, the overall value of the binding free energy (Figure 3D) levels to the reference with as few all‐atom residues as 4, the separate components oscillate and reach the plateau only for larger numbers. The existence of a minimum in the standard deviation of all three contributions pinpoints a particular number of AT active site residues for which the accuracy of the computed free energy is the highest and the economy of the high‐resolution subpart the largest. Including more than 6 AT residues counterintuitively worsens the results ‐when the various contributions are looked at‐ and the previous accuracy is only recovered when more residues are included. This behavior suggests that the total free energy undergoes an error cancelation that hides the deviations of the separate terms.

**FIGURE 5:**
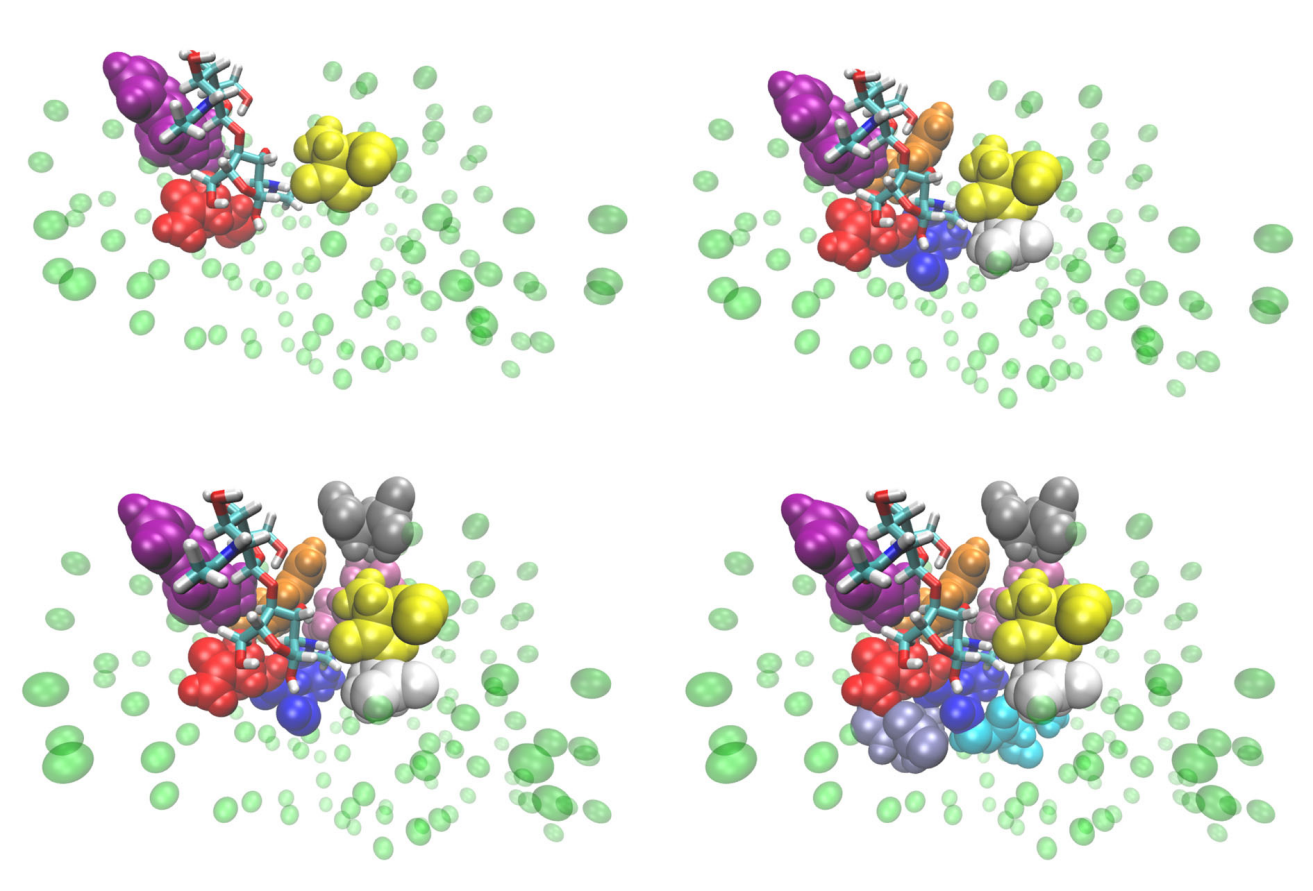
VMD representation of lysozyme and ligand in different resolution: A, 3; B, 6; C, 8; and D, 10 atomistic residues. The complete set can be found in Supporting Information. The ligand is always atomistic and it is represented in Licorice. In green are represented the elastic network model beads. With the other colors are represented, instead, the various atomistic residues that surround the ligand [Color figure can be viewed at wileyonlinelibrary.com]

**FIGURE 6:**
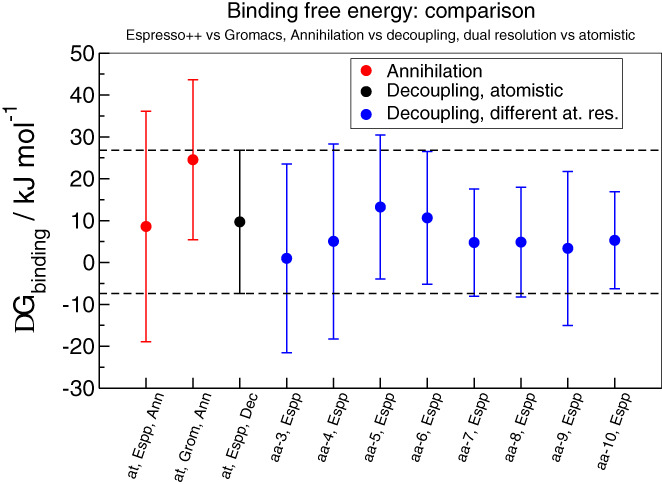
Binding free energies as a function of the number of protein residues included in atomistic detail in the multiresolution setup, as well as in a fully atomistic setup. The heavy dashed black horizontal lines and black point are the reference values from fully atomistic simulations obtained in ESPResSo++ with decoupling, and the lighter dotted black horizontal lines are the error bars for those values. Binding free energies values in ESPResSo++ and GROMACS in case of annihilation are represented in red. The binding free energy value in dual resolution simulation changing the number of atomistic residues is represented in blue [Color figure can be viewed at wileyonlinelibrary.com]

A possible explanation for this nontrivial behavior is that when 6 active site residues are modeled with all‐atom accuracy (Figure 5B) the ligand is stable in the catalytic site, namely it is surrounded by a complete shell of AT residues. The addition or deletion of other residues (Figure 5C,A, respectively) leads to a worsening of Δ*G*: in the first case, the two added residues (in pink and gray) are located behind the first shell of amino acids (far away from the ligand) and start to form a second, incomplete shell; in the second case, only three AT amino acids take part in the direct interaction with the ligand: therefore, the first layer is still incomplete and important interactions are missing; in order to improve the free energy value one has to add further amino acids in order to complete the second shell. We emphasize that the impact on the deviation from the reference is inversely proportional to the distance of the added/removed amino acid. Thus, the farther the AT amino acid is from the ligand, the more negligible its effect is. In the Supporting Information, we provide detail about the other numbers of all‐atom residues not reported here. Finally, the values of binding free energy (also for the case of annihilation whose calculations are reported in the Supporting Information) are summarized in Table 3 and illustrated in Figure 6.

**TABLE 3:** Representation of free energies values computed in ESPResSo++ and GROMACS (respectively *espp* and *grom* using a short notation on the table) in case of annihilation and decoupling

	Ligand	Complex	Binding
Annihilation			
*atom, espp*	−1275.3 ± 11.2	1315.2 ± 16.3	8.6 ± 27.5
*atom, grom*	−1259.0 ± 5.9	1314.8 ± 13.2	24.5 ± 19.1
Decoupling			
*atom, espp*	−151.9 ± 8.0	193.0 ± 9.1	9.8 ± 17.1
*aa‐3, espp*	−151.9 ± 8.0	184.2 ± 14.4	1.0 ± 22.4
*aa‐4, espp*	−151.9 ± 8.0	188.3 ± 15.3	5.1 ± 23.3
*aa‐5, espp*	−151.9 ± 8.0	196.4 ± 8.5	13.2 ± 16.5
*aa‐6, espp*	−151.9 ± 8.0	193.8 ± 7.8	10.6 ± 15.8
*aa‐7, espp*	−151.9 ± 8.0	187.9 ± 4.8	4.7 ± 12.8
*aa‐8, espp*	−151.9 ± 8.0	188.0 ± 5.1	4.8 ± 13.1
*aa‐9, espp*	−151.9 ± 8.0	186.6 ± 10.3	3.4 ± 18.3
*aa‐10, espp*	−151.9 ± 8.0	188.5 ± 3.5	5.3 ± 11.5

*Note*: The table is divided in three column: from left to right are represented the ligand, protein‐ligand complex and binding FE. The latter is the algebraic sum of Δ*G*_compl_, Δ*G*_r_off_, and Δ*G*_lig_. Results are in kJ mol^−1^.

## 4 CONCLUSIONS

In this work, we have shown how the dual resolution model employed, constituted by an all‐atom subregion coupled to an ENM remainder, can be used to calculate the binding free energy of an enzyme‐substrate complex with AT accuracy. Furthermore, and most importantly, we have highlighted the impact that different choices of the model resolution can have. Specifically, we have computed the total value of the binding free energy as well as that of its various energetic components, and quantitatively inspected how these change when different selections are performed for the subgroup of amino acids, ranging from 3 to 10 in total, to be modeled at the fully AT level.

At first sight, one can appreciate that the binding free energy value rapidly converges to the AT reference when as few as 4 amino acids constituting the active site are described all‐atom. This comforting result, however, unveils a greater complexity when the different terms constituting the free energy are looked at separately. These show an oscillating behavior as the number of all‐atom residues in the active site is increased, with a decreasing difference from the reference followed by a sudden jump to larger values, which dampens upon further addition of AT amino acids. The rationale in this behavior is identified in the structure of the active site, which is constituted by a first shell of the six residues exposed to the solvent and closest to the ligand; when further amino acids beyond these are modeled with AT resolution, they interact with the substrate affecting the binding free energy components and shifting them away from the reference, with a steadily lowering impact as the model's resolution is increased—as one can expect. Surprisingly, very little if no signal of this behavior is observed in the value of the binding free energy as a whole, rather it becomes visible only upon inspection of its separate contributions.

The results of this work thus highlight the importance of the mapping in the construction of multiscale and multiresolution models, as a higher degree of detail does not necessarily correlate with a higher accuracy of the quantities of interest. The implications of these observations should serve as a warning in the realization of CG models concurrently employing various levels of detail for different regions of the same system, whose range of application spans from fundamental understating of a molecule's properties to real‐life pharmaceutical applications.

## Supporting information


**Data S1** Supporting Information.
