# Supplementary material for: Ligand‐protein interactions in lysozyme investigated through a dual‐resolution model
Source: Proteins. 2020 Jun 15;88(10):1351–60. doi: 10.1002/prot.25954 (PMC7497117; doi:10.1002/prot.25954)
Supplement: Supplementary file 1 — Data S1 Supporting Information. [file PROT-88-1351-s001.pdf]

Supporting information for
Ligand-protein interactions in lysozyme investigated through a dual-resolution model

Raffaele Fiorentini,[1] Kurt Kremer,[1] and Raffaello Potestio[1,2,3,*]
[1] *Max Planck Institute for Polymer Research, Mainz, Germany*
[2] *Physics Department, University of Trento, via Sommarive, 14 I-38123 Trento, Italy*
[3] *INFN-TIFPA, Trento Institute for Fundamental Physics and Applications, I-38123 Trento, Italy*
(Dated: May 4, 2020)

I. THERMODYNAMIC CYCLE FOR BINDING FREE ENERGY

In order to compute the binding free energy ΔG_{bind} [1–3], we use a thermodynamic cycle which connects the protein-bound and protein-unbound ligand states through several intermediate ones as shown in the Fig.1 main.

Starting from the top-right corner we have the complex, with the ligand and protein fully interacting as in a normal MD simulation. The first step is adding a set of restraints between ligand and protein (giving ΔG_{restr_on}) in order to avoid the problem of the ligand leaving the binding pocket when interactions are being removed. The presence of restraints is indicated in the cycle scheme in the figure by a red circle, which represents the fact that the ligand is being confined to a certain volume. The set of restraints described by Boresch is used for this work [3]. These are quite useful as they restrain position and orientation of the compound relative to the protein, and they have an analytical solution for their removal.

The next step is decoupling the ligand from the system in order to get to the bottom-right corner of the cycle. This involves running a number of separate simulations at different λ values, first decoupling coulombic interactions ($\Delta G_{coul,c}$) and then Lennard-Jones ($\Delta G_{LJ,c}$).

Going up from the bottom-left corner of the cycles, the first step (ΔG_{restr_off}) is carried out analytically without need to run more simulations. At this point the ligand has come back to interact with the solvent, which means one needs to turn on charges ($\Delta G_{coul,\ell}$) and Van der Waals ($\Delta G_{LJ,\ell}$) parameters again, in order to obtain ΔG_{int_water} (or ΔG_{ligand}). Finally, at the top-left corner of the cycle, one sums up all the steps done so far to obtain the quantity ΔG_{bind}.

II. ANNIHILATION AND BINDING FREE ENERGY

The calculation of free energy can be done in two different ways: *decoupling* and *annihilation*. The difference between the two is the following: decoupling a molecular interaction refers to turning off that interaction between the molecule and its environment, whereas annihilation of a molecular interaction refers to turning off that interaction entirely.

We focus on the results of free energy in case of annihilation. This has two advantages: the first one is that it allows one to validate the implementation of protein free energy in ESPResSo++ [4, 5] doing a comparison with GROMACS [6]: this is feasible only in the case of annihilation in fully atomistic system because GROMACS cannot perform decoupling and dual resolution simulations.

The second advantage is that the simulation with annihilation allows us to give a further confirmation that the value of binding free energy in case of decoupling is correct, thereby proving the consistency between the two. By definition, in annihilation there are three components (ligand-ligand, ligand-water and ligand-protein) unlike decoupling which has two components (ligand-water and ligand-protein): hence, the values of complex and ligand free energy will be different each other, but the values of the resulting ΔG_{bind} in both cases agree each other within the error bar, as reported in the main text.

Without going into the simulation details (look in the apposite section of the article) we can see the results of binding free energy calculation by using Thermodynamic Integration (TI) [7].

A. Results of Binding free energy calculation

Recall that ΔG_{bind} consists in the algebraic sum of three terms: $\Delta G_{complex}$, ΔG_{ligand} and ΔG_{restr_off}. Let us focus, first of all, to the calculation of the latter because it is carried out analytically without needing to run simulations [3].

$$-\frac{\Delta G_{restr\_off}}{kT} = \\ = \ln\left[\frac{8\pi^2 V^0}{r_0^2 sin\theta_{A,0} sin\theta_{B,0}} \frac{(K_r K_{\theta_A} K_{\theta_B} K_{\phi_A} K_{\phi_B} K_{\phi_C})^{\frac{1}{2}}}{(2\pi kT)^3}\right] \quad (1)$$

where: k is the ideal gas constant; T is the temperature in Kelvin; V^0 is the volume corresponding to the one molar standard state (1660 $\mathring{A}^3$); r_0 is the reference distance for the restraints; θ_A,θ_B are the reference angles for the

* raffaello.potestio@unitn.it

restraints; K_x is the force constant for the distance (r_0), two angles (θ_A,θ_B) and three dihedrals (ϕ_A, ϕ_B, ϕ_C) restraints we applied.

In our case we have that:

$$k = 8.31\frac{J}{mol\cdot K} = 1.987\frac{cal}{mol\cdot K}$$
$$T = 298K$$
$$V^0 = 1660\text{Å}^3$$
$$r_0 = 0.31nm = 3.1\text{Å}$$
$$k_x = 4184\frac{KJ}{mol\cdot nm^2} = 41.84\frac{KJ}{mol\cdot \text{Å}^2}$$
$$\theta_A = 120^o$$
$$k_{\theta_A} = 41.84\frac{KJ}{mol\cdot rad^2}$$
$$\theta_B = 90^o$$
$$k_{\theta_B} = 41.84\frac{KJ}{mol\cdot rad^2}$$
$$k_{\phi_A} = k_{\phi_B} = k_{\phi_C} = 41.84\frac{KJ}{mol\cdot rad^2}$$

Therefore the contribution to the binding free energy coming from the restraints amounts to:

$$\Delta G_{restr\_off} = -31.3 \ \ kJ\cdot mol^{-1} \quad (2)$$

The results of the $\Delta G_{complex}$ and ΔG_{ligand} terms and the alchemical changes, comparing ESPResSo++ and GROMACS are shown respectively in Tab.I and Tab.II and illustrated in the Fig.1.

TABLE I: Resulting values of free energy of Complex Free Energy (4th column) and its components (Coulomb, Lennard Jones and Restraints in the first three columns) in fully atomistic system in case of annihilation. All values are in $kJ\cdot mol^{-1}$ and performed with Thermodynamic Integration. All simulations are carried out in GROMACS and ESPResSo++ (*grom* and *espp* in the table). For each value of λ, the fully atomistic simulations lasts 1 ns by using both MD package program simulation. These results show that, within the error bars, both codes provide the same results.

**Complex FE - Annihilation**				
	$\Delta G_{coul,c}$	$\Delta G_{LJ,c}$	$\Delta G_{Restr\_on,c}$	$\Delta G_{complex}$
**grom**	1254.2 ± 8.0	57.3 ± 4.9	3.3 ± 0.3	1314.8 ± 13.2
**espp**	1250.7 ± 5.6	60.8 ± 10.4	3.6 ± 0.4	1315.1 ± 16.4

The resulting Binding Free Energy is shown in the Tab. III: we take up the final values of Complex and Ligand FE, in order to compute ΔG_{bind}. These results are illustrated in the Fig.2.

These plots show how GROMACS and ESPResSo++ produce the same Binding FE results both in the components (Fig.1) and in the total (Fig.2).

TABLE II: Resulting values of Ligand Free Energy (3rd column) and its components (Coulomb, Lennard Jones in the first two columns) in fully atomistic system in case of annihilation. All the values are in $kJ\cdot mol^{-1}$ and performed with Thermodynamic Integration. All simulations are carried out in GROMACS and ESPResSo++ (*grom* and *espp* in the table). For each value of λ, the fully atomistic simulations lasts 1 ns by using both MD package program simulations.

**Ligand FE - Annihilation**			
	$\Delta G_{coul,\ell}$	$\Delta G_{LJ,\ell}$	ΔG_{ligand}
**grom**	1238.8 ± 2.3	20.2 ± 3.6	1259.0 ± 5.9
**espp**	1250.1 ± 6.2	25.2 ± 5.0	1275.3 ± 11.2

TABLE III: Representation of Free Energies values computed in ESPResSo++ and GROMACS (*espp* and *grom* respectively in the table) in case of annihilation. The table is divided in three column: from left to right are represented the ligand, protein-ligand complex and binding FE. These results are in $kJ\cdot mol^{-1}$.

**Binding FE - Annihilation**			
	ΔG_{ligand}	$\Delta G_{complex}$	$\Delta G_{binding}$
**grom**	-1259.0 ± 5.9	1314.8 ± 13.1	24.5 ± 19.1
**espp**	-1275.3 ± 11.2	1315.2 ± 16.3	8.6 ± 27.5

As reported in the main article, comparing the results of the Binding FE values both in annihilation and decoupling, we notice that there is a consistency between these two method of treating interactions (Fig. 6 main). Therefore we chose to work with decoupling instead of annihilation, but we choose the first one because this process is more intuitive with respect its annihilation turning off the interactions within it. Moreover, the ligand is always treated atomistically, therefore it is not involved in the change of free energy varying the protein resolution.

III. PARAMETRIZATION OF THE DUAL-RESOLUTION MODEL

Our protein is treated in dual-fixed-resolution, in particular the binding site of lysozyme is modelled in atomistic high level of resolution, whereas the rest of protein is treated in Coarse Grained and specifically in ENM [8].

In order to construct a good dual resolution model, the system needs a parametrization. The latter was already performed in [9]. Here we describe the key elements of the model parametrization, namely the elastic constant between consecutive ENM nodes and not consecutive ones, and the parameters ϵ and σ of Week-Chandler-Anderson (WCA) [10]. In particular, the latter was found in the case of 8 atomistic residues, therefore in this section we must confirm that it is still good changing the protein resolution.

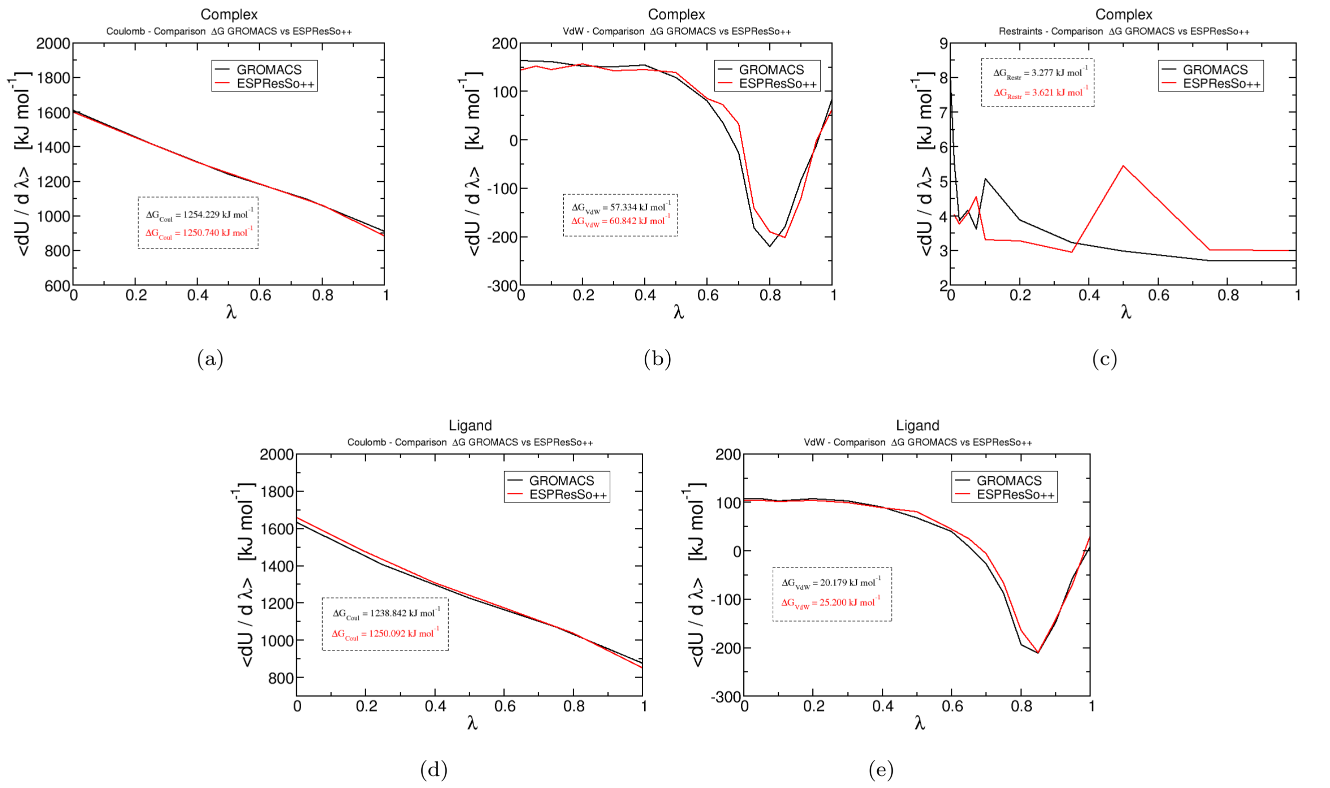

FIG. 1: Comparison of the Thermodynamic Integration (TI) free energy derivative curves computed with ESPResSo++ and GROMACS for all atom protein. (a) Coulomb, (b) Lennard-Jones and (c) restraint free energies curves for the protein-ligand complex, and (d) Coulomb and (e) Lennard-Jones free energies curves for the ligand. These simulations use annihilation.

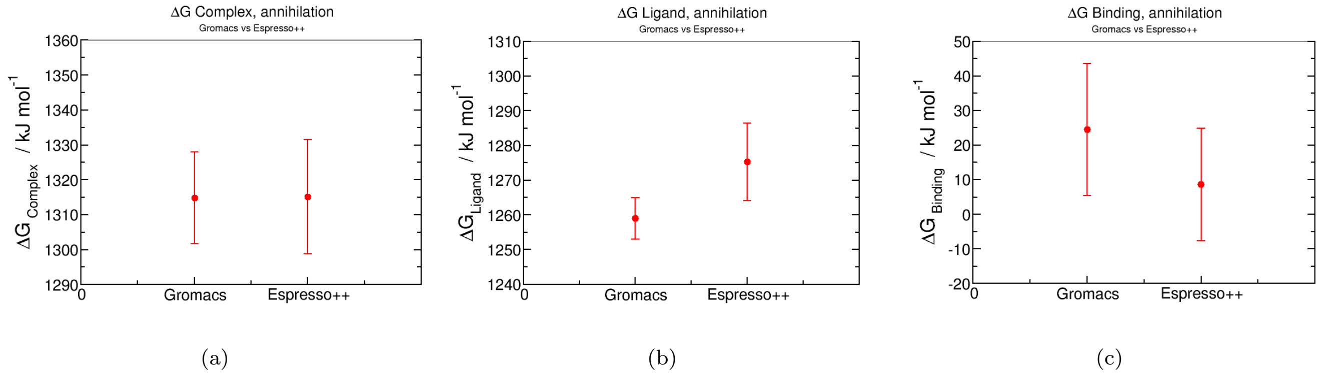

FIG. 2: Comparison of the Thermodynamic Integration (TI) free energy values computed with ESPResSo++ and Gromacs for all atom protein. (a) protein-ligand complex, (b) Ligand and (c) Binding free energy values (with error bars). These simulations use annihilation. The plots show that the results obtained via ESPResSo++ and Gromacs are comparable within the error bars.

A. Determination of elastic constants between beads

First, we do a distinction between the value of elastic constants between consecutive C_α beads along the protein backbone (k_b) and not consecutive ones (k_{nb}) until the cutoff set to 1.2 nm. In particular, we take as k_b, the stiff value of $5 \cdot 10^4$ $kJ \cdot mol^{-1} \cdot nm^{-2}$. The global fluctuations are independent of this value. All other spring constants have a value k_{nb} = 160 $kJ \cdot$

$mol^{-1} \cdot nm^{-2}$, parametrised by minimising the average root mean square error in C_α rmsf and the S^2 order parameter calculated from ENM relative to fully atomistic simulations [9].

B. Determination of WCA parameters ε and σ

When the ENM is employed in multi-resolution simulations, an excluded volume interaction between ENM nodes and solvent molecules is required, in order to prevent from penetrating the protein and solvating the atomistic binding site from the interior. Thus, a WCA interaction is applied between C_α nodes and all the solvent molecules.

In its formulation, WCA needs two parameters: ϵ and σ. The former has a value of 0.34 $kJ \cdot mol^{-1}$, arbitrarily chosen as the value for carbon in the atomistic forcefield, whilst $\sigma_i = R_{g,i} \cdot c$, where $R_{g,i}$ is the radius of gyration of a given residue i out of the twenty possible amino acids and c is the same for all amino acids. The latter is not known a priori, because its value has to be tuned to give the correct bulk water density for a protein-water system (i.e. the water density far from the protein) from fully atomistic simulation.

In order to find the proper value of c we started with the 8 atomistic residues protein launching different dual resolution simulations of 1 ns, varying its value. After finding the correct c such that the density between atomistic and dual-res system are comparable, we checked that such a value is still good launching, this time, 1 ns simulations with different numbers of atomistic residues keeping c fixed.

Tab. IV and Fig.3 show the bulk water density in the fully atomistic reference system and in case of 8 amino acids modelled atomistically for different values of c.

TABLE IV: density found in the case of 8 atomistic residues for different value of c and comparison with the atomistic reference. Each dual resolution simulation varying c lasts 1 ns.

**aa-8**	**density / molecules nm^{-3}**
fully at	100.2
0.59	99.5
0.61	99.8
0.63	99.9
0.65	100.1
0.67	100.4
0.69	100.5
0.71	100.7

In particular Fig.3 also shows a linear interpolation between points in order to get c as precise as possible. The resulting value of c is 0.658.

Tab.V shows that such a value is still valid when changing the number of amino acids of protein active site, because the relative error is no longer than 0.7%. We thus employed $c = 0.658$ in all considered cases.

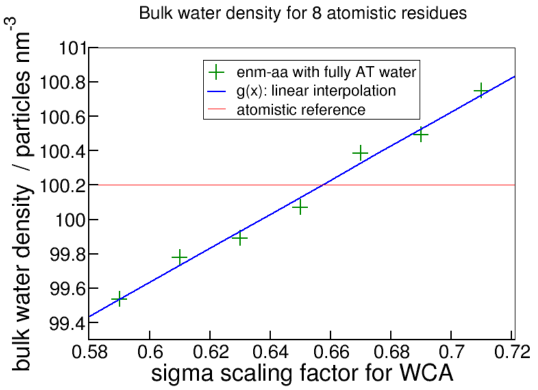

FIG. 3: Bulk water density in the case of 8 atomistic residues for different value of c. The atomistic reference value is 100.2 and it represented with the red line, whereas the linear interpolation of points is called g(x) in the legend and it is shown with a blue line.

In Fig.4 we report the VMD [11] representation of all the considered cases changing the number of atomistic residues of active site from 3 to 10 (recall that in the article are reported only the most important cases namely three, six, eight and ten atomistic residues).

TABLE V: Bulk water's average density (in $molecules \cdot nm^{-3}$) and percentage relative error in dual-resolution simulation with different atomistic residues from 3 to 10, keeping $c = 0.658$. Each simulation lasts 1 ns.

**# at residues**	**average density**	**relative error**
**3**	100.3	0.1 %
**4**	100.1	0.1 %
**5**	100.1	0.1 %
**6**	100.1	0.1 %
**7**	100.0	0.2 %
**8**	100.2	0.0 %
**9**	100.0	0.2 %
**10**	100.9	0.7 %

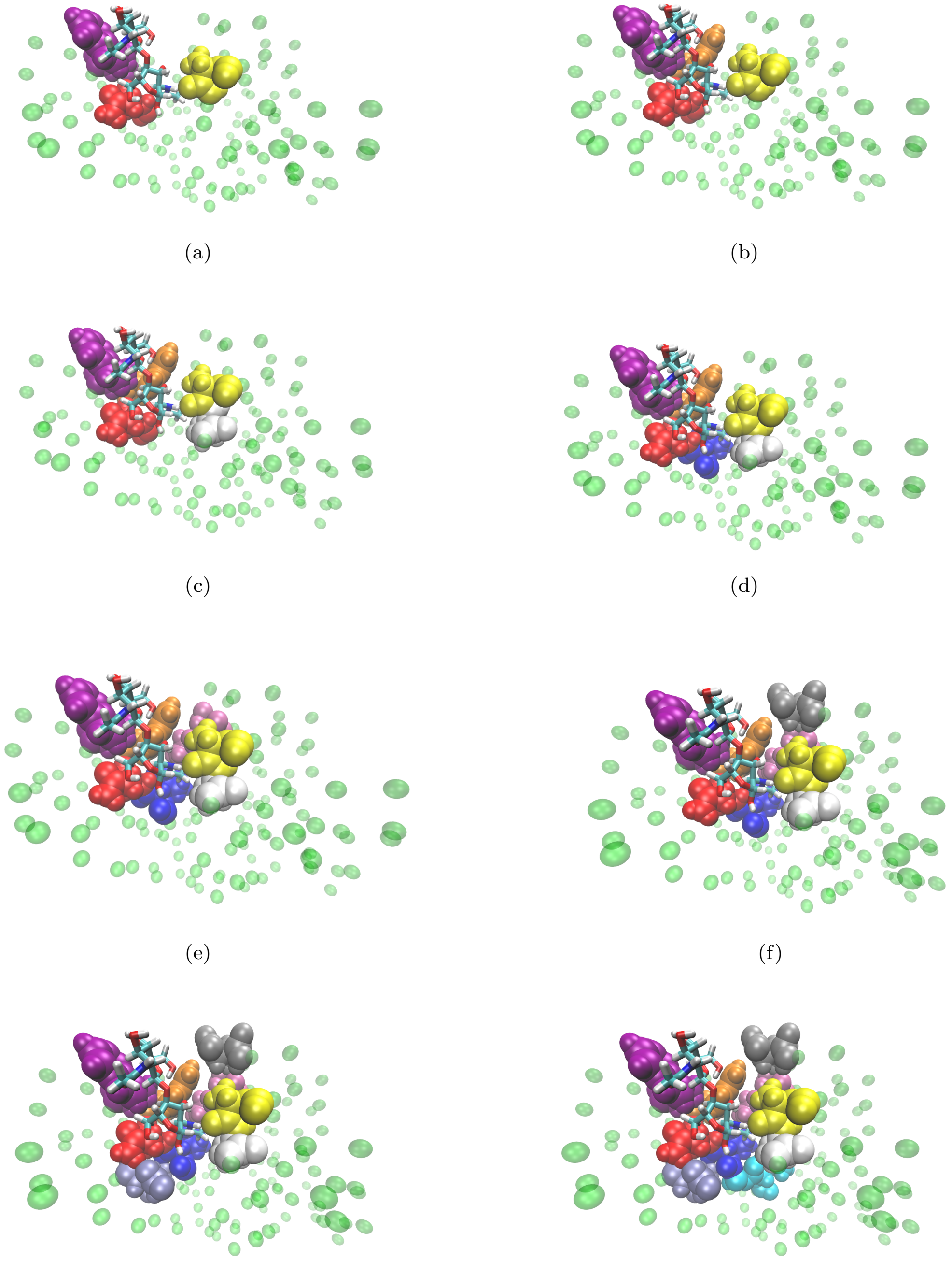

(a) (b) (c) (d) (e) (f) (g) (h)

FIG. 4: Representation of lysozyme and ligand in different resolution: (a) three, (b) four, (c) five, (d) six, (e) seven, (f) eight (g) nine, (h) ten atomistic residues. The ligand is always atomistic and it is represented in Licorice. In green are represented the ENM beads. With the other colors are represented, instead, the various atomistic residues which surround the ligand.

[1] Boyce SE, Mobley DL, Rocklin GJ, Graves AP, Dill KA, and Shoichet BK. Predicting ligand binding affinity with alchemical free energy methods in a polar model binding site. *J Mol Bio.*, page 747763, 2009.

[2] Aldeghi M., Bluck J.P., and Biggin P.C. Absolute alchemical free energy calculations for ligand binding: A beginners guide. *Computational Drug Discovery and Design*, 1762:199–232, 2018.

[3] Stefan Boresch, Franz Tettinger, Martin Leitgeb, and Martin Karplus. Absolute binding free energies: A quantitative approach for their calculation. *J. Phys. Chem. B*, 107(35):9535–9551, 2003.

[4] J.D.Halverson, T.Brandes, O.Lenz, A.Arnold, S.Bevc, V.Starchenko, K.Kremer, T.Stuehn, and D.Reith. Espresso++: A modern multiscale simulation package for soft matter systems. *Computer Physics Communications*, 184:1129–1149, 2013.

[5] Horacio V. Guzman, Nikita Tretyakov, Hideki Kobayashi, Aoife C. Fogarty, Karsten Kreis, Jakub Krajniak, Christoph Junghans, Kurt Kremer, and Torsten Stuehn. Espresso++ 2.0: Advanced methods for multiscale molecular simulation. *Computer Physics Communications*, 238:66 – 76, 2019.

[6] Berk Hess, Carsten Kutzner, David van der Spoel, and Erik Lindahl. Gromacs 4: Algorithms for highly efficient, load-balanced, and scalable molecular simulation. *Journal of Chemical Theory and Computation*, 4(3):435–447, 2008. PMID: 26620784.

[7] John G. Kirkwood. Statistical mechanics of fluid mixtures. *The Journal of Chemical Physics*, 3(5):300–313, 1935.

[8] Monique M. Tirion. Large amplitude elastic motions in proteins from a single-parameter, atomic analysis. *Phys. Rev. Lett.*, 77:1905–1908, Aug 1996.

[9] Aoife C. Fogarty, Raffaello Potestio, and Kurt Kremer. A multi-resolution model to capture both global fluctuations of an enzyme and molecular recognition in the ligand-binding site. *Proteins: Struct., Func., and Bioinf.*, 84(12):1902–1913, 2016.

[10] John D. Weeks, David Chandler, and Hans C. Andersen. Role of repulsive forces in determining the equilibrium structure of simple liquids. *The Journal of Chemical Physics*, 54(12):5237–5247, 1971.

[11] William Humphrey, Andrew Dalke, and Klaus Schulten. VMD – Visual Molecular Dynamics. *Journal of Molecular Graphics*, 14:33–38, 1996.
